# Lidocaine intensifies the anti-osteogenic effect on inflammation-induced human dental pulp stem cells via mitogen-activated protein kinase inhibition

**DOI:** 10.1016/j.jds.2022.11.020

**Published:** 2022-12-01

**Authors:** Sang-Hoon Lee, Cheul-Hong Kim, Ji-Young Yoon, Eun-Ji Choi, Mi Kyoung Kim, Ji-Uk Yoon, Hee Young Kim, Eun-Jung Kim

**Affiliations:** a Department of Dental Anesthesia and Pain Medicine, School of Dentistry, Pusan National University, Dental Research Institute, Yangsan, Republic of Korea; b Dental and Life Science Institute, School of Dentistry, Pusan National University, Yangsan, Republic of Korea; c Department of Anesthesia and Pain Medicine, School of Medicine, Pusan National University, Yangsan, Republic of Korea

**Keywords:** Dental pulp stem cell, Inflammation, Lidocaine, Mitogen-activated protein kinases, Osteogenic differentiation

## Abstract

**Background/purpose:**

Human dental pulp stem cells (hDPSCs) are an emerging source of mesenchymal stem cells (MSCs) for bone tissue regeneration and engineering. In bone regeneration using transplanted MSCs, the extracellular environment or co-injected drugs can affect their success or failure. In this study, we investigated the effects and signaling mechanisms of lidocaine on osteogenic differentiation of hDPSCs after inducing inflammatory conditions with lipopolysaccharide (LPS) and tumor necrosis factor-alpha (TNF-α).

**Materials and methods:**

To investigate the effect of lidocaine on the osteogenic differentiation of LPS/TNF-α-treated hDPSCs, alkaline phosphatase (ALP) and Alizarin red S (ARS) staining were conducted. The expression of osteogenesis-related genes was assessed using quantitative real-time polymerase chain reaction and western blotting. The expression of mitogen-activated protein kinases was analyzed to evaluate the effect of lidocaine on osteogenic differentiation of LPS/TNF-α-treated hDPSCs.

**Results:**

Various concentrations of lidocaine (0.05, 0.2, and 1 mM) further decreased ALP and ARS staining of LPS/TNF-α-treated hDPSCs. Similarly, the mRNA and protein expression of osteogenesis-related genes was suppressed via lidocaine treatment in LPS/TNF-α-treated hDPSCs. Lidocaine treatment downregulated the protein expression of p-ERK and p-JNK in LPS/TNF-α-treated hDPSCs.

**Conclusion:**

Lidocaine intensified the inhibition of osteogenic differentiation on inflammation-induced hDPSCs by inhibiting the ERK and JNK signaling pathways. This in vitro study suggested that lidocaine may have an inhibitory effect on bone regeneration.

## Introduction

In the field of tissue engineering and regenerative medicine, mesenchymal stem cells (MSCs) are gaining attention as a therapeutic tool for bone regeneration during various clinical bone loss conditions, such as osteoporosis, osteomyelitis, osteonecrosis, fracture, and bone defects.^1^ MSCs are isolated from various tissues, such as the bone marrow, adipose tissue, umbilical cord, and dental pulp.^2^^,^^3^ Among them, bone marrow–derived MSCs have been most widely used for bone regeneration, although the process of collection of MSCs from bone marrow is an invasive and painful procedure.^4^ Whereas, human dental pulp stem cells (hDPSCs) can be collected from discarded premolars or third molars, and the collection process is easy and minimally invasive.^5^ hDPSCs have many advantages, such as long in vitro survival time, high proliferation rate, and low immunosuppressive effect, compared to bone marrow–derived MSCs. In addition, several studies have reported that hDPSCs induce osteogenesis and bone regeneration similar to bone marrow–derived MSCs.^6^, ^7^, ^8^ Therefore, the use of hDPSCs for in vitro and in vivo experiments associated with bone tissue regeneration is increasing.

For bone tissue engineering and regenerative medicine, various MSCs have been transplanted into diseased or damaged bone tissues via local injection or surgical implantation with bone tissue engineering scaffolds.^9^^,^^10^ The survival rate and regeneration capacity of transplanted MSCs are influenced by various factors, including bone defect size, tissue damage, bacterial infection, inflammation, and co-injectable drugs.^11^^,^^12^ Local anesthetics (LAs) are commonly used for pain treatment in various clinical fields such as regional anesthesia, local infiltration, and nerve blocking.^13^^,^^14^ During cartilage repair operations in orthopedic surgery, intra-articular administration of human MSCs is often performed in the regions surrounding the damaged ligament or tendon, accompanied by intra-articular injection of LAs.^15^^,^^16^ Therefore, it is important to elucidate the effects of LAs on the differentiation and regeneration capacities of MSCs for successful MSC-based regenerative therapy. Previous studies have reported that LAs have cytotoxic effects on various types of MSCs.^17^^,^^18^ However, these studies have some limitations, such as small sample size, heterogeneity of study design, and few studies on the effect of LAs on the cytotoxicity and osteogenic differentiation of hDPSCs.^12^

Inflammation can affect the osteogenic differentiation of MSCs.^19^^,^^20^ Lipopolysaccharide (LPS), an outer membrane component of gram-negative bacterial cells, is an important pathogenic factor in alveolar bone resorption and can suppress osteogenic differentiation of hDPSCs.^21^^,^^22^ In addition, a previous report stated that tumor necrosis factor-alpha (TNF-α), a proinflammatory cytokine, can impair osteogenic differentiation of hDPSCs.^23^ Lidocaine is the most widely used amide-type LAs, is known to have anti-inflammatory effects in many experimental studies, and has a cytotoxic effect on MSCs.^24^^,^^25^ These findings raise the question of how lidocaine influences osteogenic differentiation of hDPSCs induced by an inflammatory response, and there is no research yet to clarify this question.

In this study, we aimed to investigate the effect of lidocaine on the osteogenic differentiation of hDPSCs after inflammatory stimulation with LPS and TNF-α. We also investigated the signaling mechanisms related to the effect of lidocaine on osteogenic differentiation of LPS/TNF-α-treated hDPSCs.

## Materials and methods

### Chemicals and reagents

A 2% lidocaine HCL injection was purchased from Jeil Pharmaceutical (Daegu, Republic of Korea). LPS was obtained from Sigma-Aldrich (St. Louis, MO, USA) and recombinant human TNF-α was purchased from PeproTech (Cranbury, NJ, USA). Αlpha Modified Eagle's medium (α-MEM), fetal bovine serum (FBS), and penicillin/streptomycin were purchased from Gibco BRL Co. (Grand Island, NY, USA). Β-glycerophosphate, ascorbic acid, dexamethasone, and Alizarin red S (ARS) were obtained from Sigma-Aldrich. StemTAG™ Alkaline phosphatase (ALP) staining kit CBA-300 was purchased from Cell Biolabs, Inc. (San Diego, CA, USA). Primary antibodies against ALP (sc-27431), OPN (sc-21742), DMP1 (sc-73633), and actin (sc47778) were purchased from Santa Cruz Biotechnology (Santa Cruz, CA, USA). Anti- BMP2 (ab14933) antibodies were purchased from abcam (Cambridge, UK). Anti-Runx2 antibody was purchased from MBL (Woburn, MA, USA). Antibodies of extracellular signal regulated kinase (ERK, 9102S), p-ERK (9101S), c-Jun N-terminal kinase (JNK, 9252S), p-JNK (9251S), p38 (9218), and p-p38 (9211) were obtained from Cell Signaling Technology (Beverly, MA, USA).

### Cell culture

hDPSCs (PT-5025, Lonza, Basel, Switzerland) were cultured in α-MEM supplemented with 10% FBS, 100 U/mL penicillin, and 100 mg/mL streptomycin, and maintained in a 5% CO_2_ incubator at 37 °C. For osteogenic differentiation, hDPSCs were cultured in osteogenic differentiation medium (ODM; α-MEM containing 10 mM β-glycerophosphate, 100 μM ascorbic acid, and 100 nM dexamethasone) for 4, 7, 14, and 21 days. For the control group, hDPSCs were cultured in the growth medium. ODM was changed every 2 days.

### 3-(4,5-dimethylthiazol)-2,5-diphenyl tetrazolium bromide (MTT) assay

To examine the effect of lidocaine treatment on cell viability and proliferation, hDPSCs were seeded in 24-well plates and treated with different concentrations of lidocaine (0, 0.05, 0.2, and 1 mM) and/or LPS (1 ng/mL)/TNF-α (10 ng/mL) for 1, 2, and 3 days. At the end of the culture period, the cells were placed in fresh medium containing 0.5 mg/mL of MTT solution and incubated for 4 h before the addition of 200 μL DMSO. The resultant blue formazan products in the hDPSCs were analyzed using a microplate reader at a wavelength of 570 nm.

### Alkaline phosphatase (ALP) staining

hDPSCs were seeded in 48-well culture plates (5 × 10^4^ cells/well) and incubated for 1 day. Cells were treated with lidocaine (0, 0.05, 0.2, and 1 mM) and LPS (1 ng/mL)/TNF-α (10 ng/mL) in control medium or ODM, and then cultured for 4 and 7 days. The culture medium was replaced with fresh medium every 2 days. ALP staining was performed 4 and 7 days after induction using a CBA-300 ALP staining kit, according to the manufacturer's protocol. Briefly, cultured cells were fixed in 4% paraformaldehyde, washed three times with PBS containing 0.05% Tween-20, and incubated with ALP staining solution for 15–30 min. Next, the ALP solution was removed, the cells were washed with PBS, and the ALP staining cells were observed under a microscope at a magnification.

### Alizarin red S (ARS) staining

After 14 and 21 days of culture, the hDPSCs were washed twice with PBS and fixed in 4% paraformaldehyde for 15 min. Before cell staining, the fixative was removed and the cells were washed three times with distilled water. The cells were then stained with a 2% ARS staining solution (pH 4.2) for 10 min at room temperature. The ARS staining solution was removed and the cells were washed with distilled water. The cells were imaged using a digital camera, and the intensity of staining was quantified using the ImageJ program (NIH, Bethesda, MD, USA).

### RNA extraction and quantitative real-time polymerase chain reaction (PCR)

Total RNA from hDPSCs was isolated using the RiboEx reagent (GeneAll, Seoul, Korea), as described by the manufacturer. One microgram of total RNA was reverse-transcribed into cDNA using a HiSenScript™ RH [-] RT PreMix Kit (INtRON Biotechnology, Seongnam, Korea). The synthesis conditions involved one cycle of 60 min at 42 °C. cDNA synthesis was inactivated by heating the sample for 10 min at 85 °C. Real-time PCR was performed using SYBR Green Q-PCR Master Mix with Low Rox kit (Applied Biosystems, Foster City, CA, USA) for 40 cycles of 15 s denaturation at 95 °C and 1 min amplification at 60 °C for all genes in the QuantStudio 1 real-time PCR system (Thermo Fisher Scientific, Waltham, MA, USA). The data are representative of three independent experiments, and the relative mRNA expression was determined using the comparative Ct (ΔΔCt) method. GAPDH was used as the reference gene for normalization. The primer sets used for the real-time PCR are listed in Table 1.

**Table 1:** Primer sequences used for the real-time PCR.

Gene	Forward primer	Reverse primer
ALP	GGACGCTGGGAAATCTGTG	CCATGATCACGTCAATGTCC
BMP2	TTCCACCATGAAGAATCTTTGG	AAACCTGAAGCTCTGCTGAG
DMP1	TAGGAAGTCTCGCATCTCAG	CCAGTGTCTCTGGAGTTGC
Runx2	TCCCAGTATGAGAGTAGGTGTCC	GGCTCAGATAAGAGGGGTAAGAC
OPN	GCAACCGAAGTTTTCACTCC	ATCAGGGTACTGGATGTCAG
GAPDH	TATGACTCTACCCACGGCAAGT	ATACTCAGCACCAGCATCACC

Abbreviations: ALP, alkaline phosphatase; BMP2, bone morphogenetic protein 2; DMP1, dentin matrix protein 1; Runx2, runt-related transcription factor 2; OPN, osteopontin; GAPDH, glyceraldehyde 3-phosphate dehydrogenase.

### Western blot

Cells were treated with a passive lysis buffer (Promega, Madison, WI, USA), followed by gentle sonication, and incubated for 30 min on ice. The supernatants were collected after centrifugation at 13,000 rpm at 4 °C for 10 min. Protein concentration in the lysates was determined using the Bradford protein assay kit (Bio-Rad Laboratories, Hercules, CA, USA). Proteins were mixed with the sample loading buffer and incubated at 100 °C for 5 min. Twenty micrograms of protein was loaded per lane, separated using 8% and 10% polyacrylamide gels, and transferred to a polyvinylidene fluoride membrane (Millipore, Billerica, MA, USA). The blots were blocked with 5% nonfat milk for 30 min at room temperature. Membranes were incubated overnight at 4 °C with the appropriate primary antibodies. The membranes were washed thrice for 10 min using phosphate-buffered saline with Tween-20 (PBST). The membranes were then incubated with horseradish peroxidase-conjugated appropriate secondary antibodies for 60 min at room temperature, and washed with PBST thereafter. The membranes were treated with the enhanced chemiluminescence solution (Millipore) and chemiluminescence was detected using the LAS4000 (GE Healthcare Life Sciences, Buckinghamshire, UK). Actin expression was used as a control. The target protein bands were normalized relative to the control band using the ImageJ software (NIH).

### Statistics

Values are presented as the mean ± standard error of the mean. The data were obtained from at least three independent experiments. Student's t-test was used to determine the significance of the differences between the two groups. Statistical significance was set at *P* < 0.05.

## Results

### Effect of LPS/TNF-α and lidocaine treatment on cell viability and proliferation of hDPSCs

**Figure 1:**
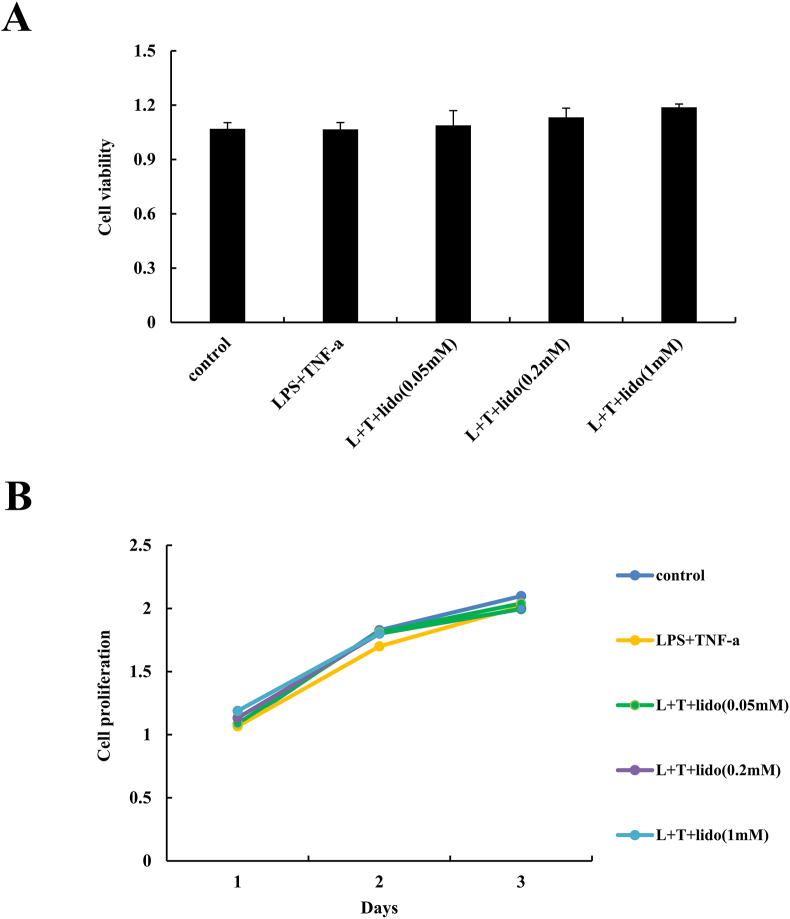
Effect of various concentrations of lidocaine (0, 0.05, 0.2, and 1 mM) and/or LPS (1 ng/mL)/TNF-α (10 ng/mL) on cell viability (A) and proliferation (B) of human dental pulp stem cells (hDPSCs) after 1, 2, and 3 days was evaluated using MTT assay.

As shown in Fig. 1A, the results of the MTT assay showed that there was no cytotoxic effect of LPS/TNF-α and lidocaine (0.05, 0.2, and 1 mM) treatment on hDPSCs. Moreover, there was no significant difference in the proliferation of hDPSCs among lidocaine (0.05, 0.2, and 1 mM) with LPS/TNF-α treatments until day 3 (Fig. 1B).

### Lidocaine has a negative effect on the osteogenic differentiation of LPS/TNF-α-treated hDPSCs

**Figure 2:**
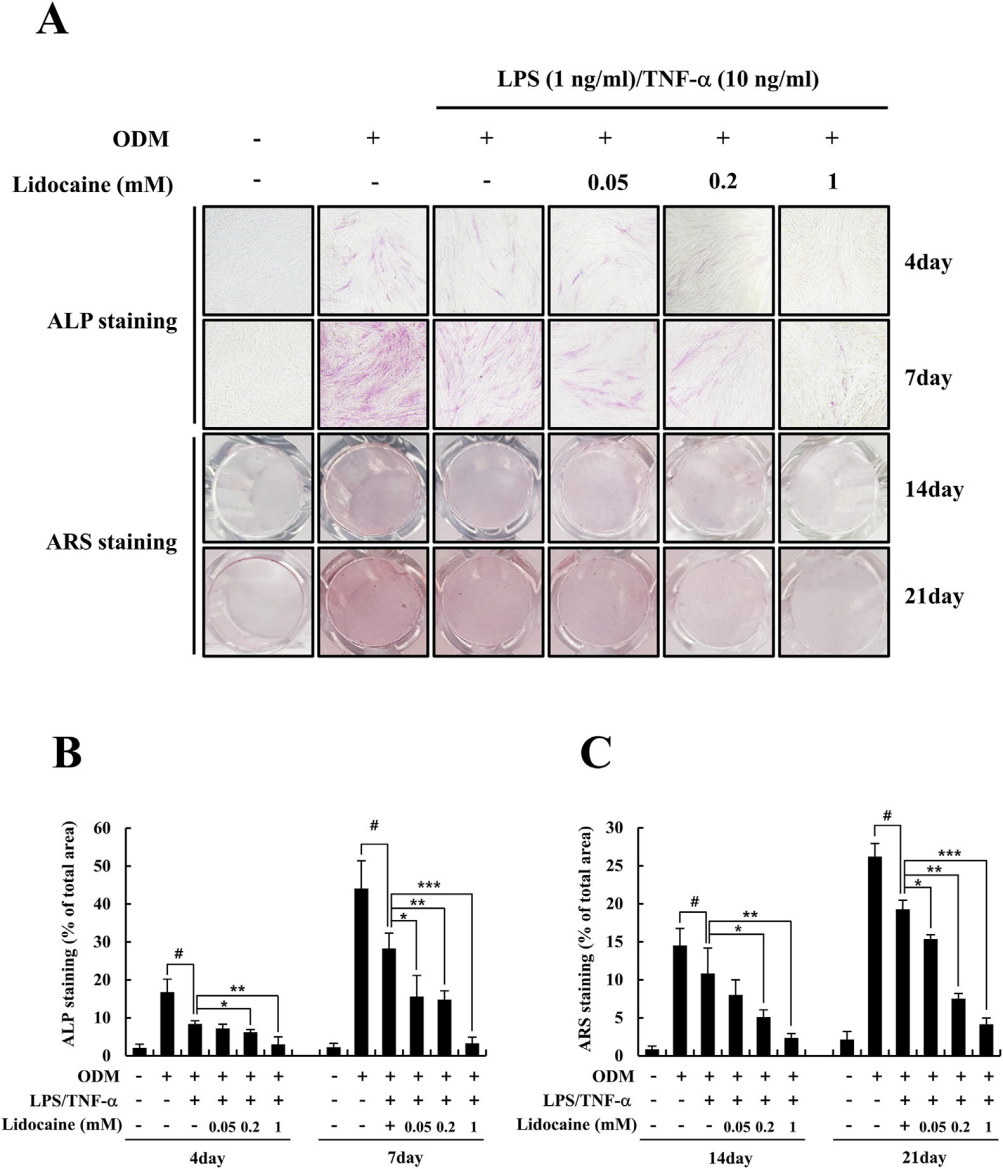
Effect of lidocaine on osteogenic differentiation in LPS/TNF-α-treated human dental pulp stem cells (hDPSCs). (A) The hDPSCs were treated with the indicated dose of lidocaine (0, 0.05, 0.2, and 1 mM) and LPS (1 ng/mL)/TNF-α (10 ng/mL) in control media or osteogenic differentiation media (ODM) for 4 and 7 days or 14 and 21 days. The cells were then fixed and stained for alkaline phosphatase (ALP) staining (after 4 and 7 days) or Alizarin red S (ARS) staining (after 14 and 21 days). Representative ALP (upper) and ARS (lower) stained images are shown. (B–C) The intensity of ALP and ARS staining was quantified. Results are presented as percentage of total area. ^#^*P* < 0.05 versus ODM group, ∗*P* < 0.05, ∗∗*P* < 0.01, ∗∗∗*P* < 0.001 versus LPS/TNF-α group.

To investigate the effect of lidocaine and/or LPS (1 ng/mL)/TNF-α (10 ng/mL) treatment on the osteogenic differentiation potential of hDPSCs, we conducted ALP and ARS staining. For osteogenic differentiation, hDPSCs were cultured in ODM for 7 days, and ALP staining was performed after 4 and 7 days. In the current study, LPS/TNF-α treatment significantly decreased ALP staining compared to the ODM group after 4 and 7 days, and all concentrations of lidocaine (0.05, 0.2, and 1 mM) significantly intensified the inhibitory effect of LPS/TNF-α on ALP staining after 7 days (Fig. 2A and B). In particular, the significant decrease in ALP staining was even greater when hDPSCs treated with LPS/TNF-α were treated with 1 mM lidocaine (*P* < 0.001). ARS staining was conducted after 14 and 21 days to examine the formation of mineralized calcium nodules related to osteogenic differentiation of hDPSCs. ARS staining showed that LPS/TNF-α treatment led to a significant decrease in mineralized calcium nodules compared with the ODM group after 14 and 21 days. This reduction was induced by lidocaine treatment in a dose-dependent manner compared to LPS/TNF-α group after 14 and 21 days (Fig. 2A, C). The reduction in ARS staining was most remarkable when LPS/TNF-α-treated hDPSCs were treated with 1 mM lidocaine for 21 days (*P* < 0.001). Therefore, LPS/TNF-α-treated hDPSCs were treated with 1 mM lidocaine for subsequent experiments.

### Lidocaine treatment suppressed the mRNA expression of osteogenesis-related genes in LPS/TNF-α-treated hDPSCs

**Figure 3:**
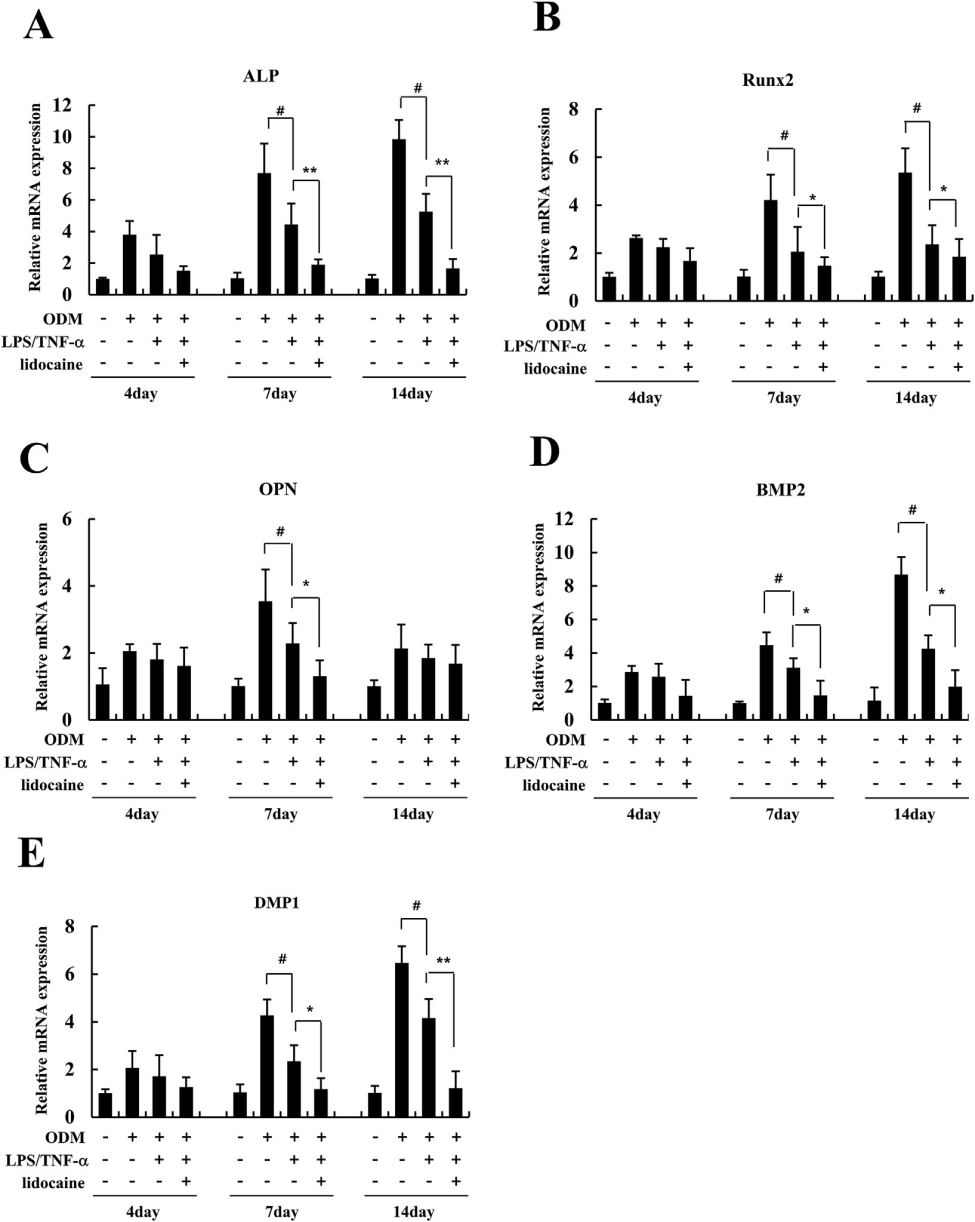
Effect of lidocaine on the mRNA expression of osteogenesis-related genes in LPS/TNF-α-treated human dental pulp stem cells (hDPSCs). The hDPSCs were treated with lidocaine (1 mM) and LPS (1 ng/mL)/TNF-α (10 ng/mL) in control media or osteogenic differentiation media (ODM) for 4, 7 and 14 days. Total RNA was isolated during osteogenic differentiation and analyzed using quantitative real-time polymerase chain reaction (PCR). The expression levels of human (A) ALP, (B) Runx2, (C) OPN, (D) BMP2, and (E) DMP1 genes were determined using real-time PCR. The expression levels in the control media were considered to be 1.0, and the values were normalized to GAPDH mRNA level. Data are expressed as mean ± standard deviation of three independent experiments in triplicate. ^#^*P* < 0.05 versus ODM group, *∗P* < 0.05*, ∗∗P* < 0.01 versus LPS/TNF-α group.

Next, we investigated the effect of lidocaine treatment on the mRNA expression of osteogenesis-related genes during the osteogenic differentiation of LPS/TNF-α-treated hDPSCs. The mRNA expression levels of ALP, Runx2, OPN, BMP2, and DMP1 were significantly upregulated in the ODM group compared to those in the control group. The mRNA expression level was elevated after 7 and 14 days, but the degree of increase was highest after 14 days, except for OPN (Fig. 3). LPS/TNF-α treatment significantly downregulated the mRNA expression of ALP, Runx2, OPN, BMP2, and DMP1 compared with the ODM group, and the downregulation of mRNA expression was strengthened using lidocaine treatment (1 mM). Downregulation of ALP, Runx2, BMP2, and DMP1 mRNA expression was significantly strengthened via lidocaine after 7 and 14 days (Fig. 3A, B, D, E). A significant downregulation of OPN mRNA expression via lidocaine was observed after 7 days (Fig. 3C).

### Protein expression of osteogenesis-related genes was suppressed by lidocaine treatment in LPS/TNF-α-treated hDPSCs

**Figure 4:**
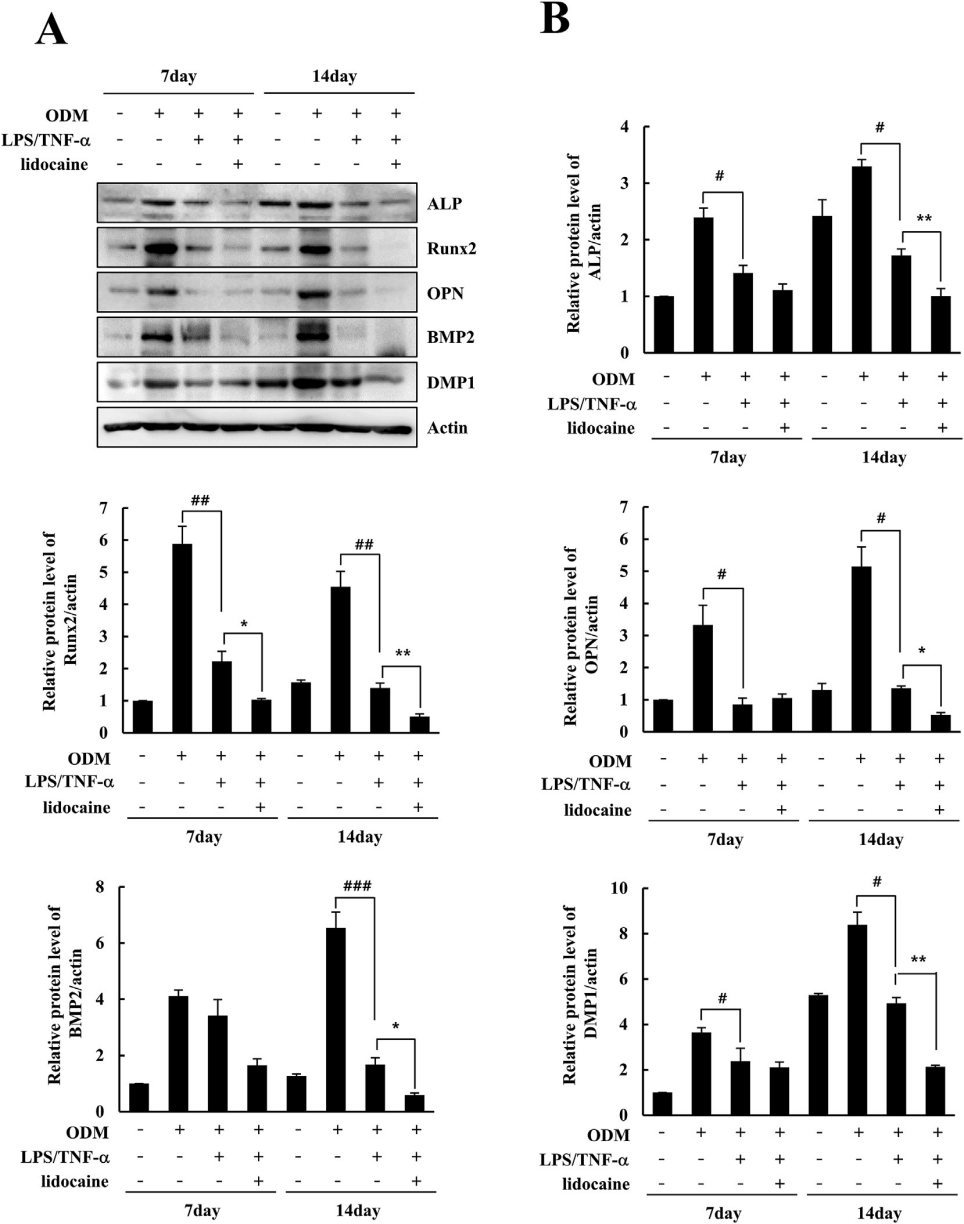
Effect of lidocaine on the protein expression of osteogenesis-related genes in LPS/TNF-α-treated human dental pulp stem cells (hDPSCs). (A) The hDPSCs were treated with lidocaine (1 mM) and LPS (1 ng/mL)/TNF-α (10 ng/mL) in control media or osteogenic differentiation media (ODM) for 7 and 14 days, and the protein expression of ALP, Runx2, OPN, BMP2, and DMP1 was examined using Western blot. (B) The expression levels in the control media were considered to be 1.0, and the values were normalized to actin protein level. Data are expressed as mean ± standard deviation of three independent experiments in triplicate. ^#^*P* < 0.05, ^##^*P* < 0.01, ^###^*P* < 0.001 versus ODM group, *∗P* < 0.05, *∗∗P* < 0.01 versus LPS/TNF-α group.

The effect of lidocaine treatment on the protein expression of osteogenesis-related genes in hDPSCs was analyzed using western blotting after 7 and 14 days. As shown in Fig. 4A and B, the protein expression levels of ALP, Runx2, OPN, BMP2, and DMP1 were significantly decreased using LPS/TNF-α treatment compared to those in the ODM group after 14 days. The 1 mM lidocaine treatment significantly decreased the protein expression of osteogenic-related genes compared to that in the LPS/TNF-α group in hDPSCs after 14 days.

### Lidocaine inhibited the activation of ERK and JNK signaling pathways in hDPSCs

**Figure 5:**
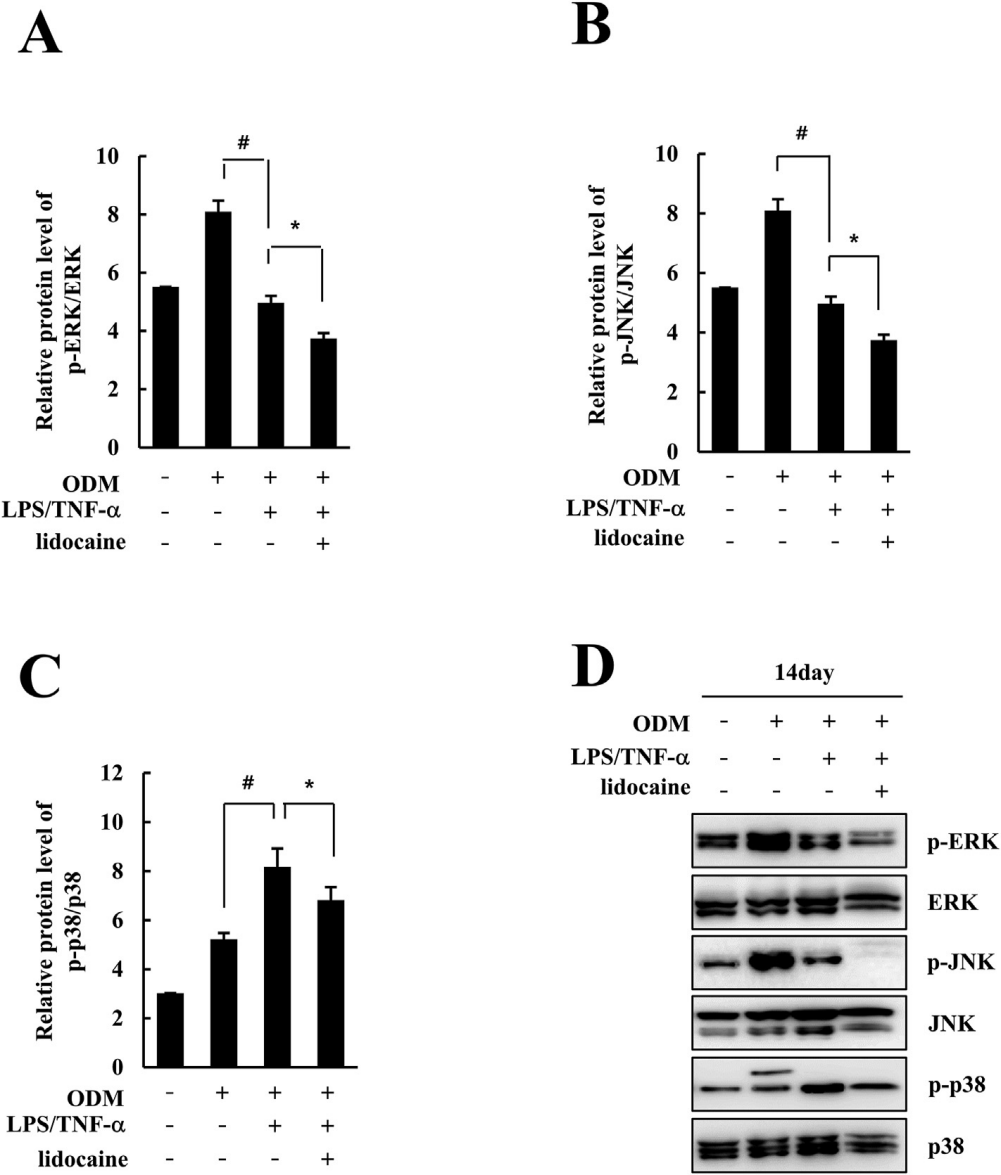
Effect of lidocaine on the expression of mitogen-activated protein kinase pathways in LPS/TNF-α-treated human dental pulp stem cells (hDPSCs) during osteogenesis. The hDPSCs were treated with lidocaine (1 mM) and LPS (1 ng/mL)/TNF-α (10 ng/mL) in control media or osteogenic differentiation media (ODM) for 14 days. The proteins were extracted and subjected to Western blot. (A–D) Immunoblots were developed using antibodies directed against p-ERK, p-JNK, and p-p38. ERK, JNK, and p38 were used as controls. All Western blot analyses were repeated three times. Band densitometry quantified by fluorChem 8900. ^#^*P* < 0.05 versus ODM group, *∗P* < 0.05 versus LPS/TNF-α group.

To investigate the role of mitogen-activated protein kinase (MAPK) pathways in the inhibitory effect of lidocaine on the osteogenic differentiation of LPS/TNF-α-treated hDPSCs, the protein expression of p-ERK, ERK, p-JNK, JNK, p-p38, and p38 was analyzed using western blotting. During the osteogenic differentiation of hDPSCs with ODM, the relative protein levels of p-ERK/ERK and p-JNK/JNK were significantly decreased using LPS/TNF-α treatment compared to those in the ODM group. In addition, 1 mM lidocaine treatment on LPS/TNF-α-treated hDPSCs significantly reduced the relative protein levels of p-ERK/ERK and p-JNK/JNK compared to those in LPS/TNF-α group of hDPSCs (Fig. 5A and B). These results were also demonstrated by the downregulation of p-ERK and p-JNK, as shown in Fig. 5D. As shown in Fig. 5C and D, lidocaine treatment significantly reduced the expression of p-p38 and relative protein levels of p-p38/p38 compared to those in LPS/TNF-α-treated hDPSCs, whereas the relative protein level of p-p38/p38 was significantly increased by LPS/TNF-α treatment compared to that in the ODM group.

## Discussion

In this study, we aimed to evaluate the effects of lidocaine on osteogenic differentiation of LPS/TNF-α-treated hDPSCs. The main findings of our experiments were as follows: (1) LPS (1 ng/mL)/TNF-α (10 ng/mL) treatment had no cytotoxic effect on hDPSCs. Lidocaine treatment (0.05, 0.2, and 1 mM) along with LPS/TNF-α was also not cytotoxic to hDPSCs. (2) Inflammatory stimulation with LPS/TNF-α suppresses osteogenic differentiation of hDPSCs. Lidocaine treatment of LPS/TNF-α-treated hDPSCs amplified the inhibitory effect of LPS/TNF-α on osteogenic differentiation, thereby further diminishing the osteogenic differentiation of hDPSCs. (3) Lidocaine treatment decreases the phosphorylation of ERK and JNK in LPS/TNF-α-treated hDPSCs. This suggests that the inhibitory effect of lidocaine on the osteogenic differentiation of LPS/TNF-α-treated hDPSCs is mediated via the suppression of the ERK and JNK pathways.

Several studies have identified the inhibitory effect of LPS or TNF-α on the osteogenic differentiation of MSCs and their associated signaling pathways.^21^, ^22^, ^23^ In vitro and in vivo studies by Li et al. reported that LPS impaired the osteogenic differentiation of human periodontal ligament stem cells via nuclear factor-kappa B (NF-κB) activation and increased alveolar bone loss.^26^ A study by Yuan et al. showed that administration of LPS decreased the osteogenic differential potential of DPSCs via activation of the HMGA2/PI3K/Akt signaling pathway.^21^ In addition, previous research has shown that TNF-α suppresses osteogenic differentiation of rat follicle stem cells through activation of the Wnt signaling pathway.^27^ However, some studies have reported the stimulatory effect of LPS or TNF-α on the osteogenic differentiation of MSCs.^28^, ^29^, ^30^ Therefore, the effect of LPS or TNF-α on the osteogenic differentiation of MSCs is still debated, and the signaling pathways involved remain unclear. To clarify the effect of LPS and TNF-α on the osteogenic differentiation of MSCs and to strengthen the inflammatory stimulation on DPSCs, we used LPS/TNF-α for the inflammatory stimulation on the DPSCs.^30^ Our results show that LPS/TNF-α suppressed the osteogenic differentiation of hDPSCs.

In bone development and homeostasis, MAPKs play a critical role in the proliferation, differentiation, and apoptosis of MSCs, particularly in osteogenic differentiation.^31^^,^^32^ Here, we found that the inhibitory effect of lidocaine on the osteogenic differentiation of LPS/TNF-α-treated hDPSCs was mediated by the inhibition of the ERK and JNK signaling pathways. Several studies have shown a correlation between ERK activation and increased bone mineralization using MSCs, calvarial osteoblasts, and in vivo bone models.^33^^,^^34^ ERK is an upstream stimulator of Runx2, a critical regulator of the osteogenic differentiation of MSCs, and directly phosphorylates Runx2.^35^ Activated JNKs catalyze the phosphorylation of several substrates for the self-renewal and differentiation of various types of stem cells.^36^ Additionally, it has been reported that JNK activates AP-1 and ATF2, which are transcription factors involved in bone formation, and inhibition of JNK suppressed the late-stage osteoblast differentiation.^37^^,^^38^ The activation of p38 is also known to play a relevant role in the osteogenic differentiation of MSCs.^39^ In the present study, even if the phosphorylation of p38 was significantly decreased using lidocaine treatment in hDPSCs compared to the LPS/TNF-α group, the protein expression of p-p38 was increased using LPS/TNF-α treatment, which is inconsistent with the fact that LPS/TNF-α suppressed the osteogenic differentiation of hDPSCs and lidocaine amplified the inhibitory effect of LPS/TNF-α on the osteogenic differentiation of hDPSCs. Therefore, we concluded that the inhibitory effect of lidocaine and LPS/TNF-α on osteogenic differentiation of hDPSCs was not mediated through inhibition of the p38 pathway.

In recent years, several studies have demonstrated that lidocaine has anti-inflammatory effects in various inflammatory models.^24^^,^^40^, ^41^, ^42^, ^43^, ^44^ Chen et al. showed that lidocaine ameliorated LPS-induced lung injury by inhibiting inflammation, which was mediated through suppression of the NF-κB and p38 signaling pathways.^40^ Zang et al. also reported that lidocaine inhibited the activation of the ERK/NF-κB pathway and led to the inhibition of inflammatory responses in a rat model.^43^ Previous studies have reported that lidocaine inhibits inflammatory effects through the inhibition of MAPK pathways. The cytotoxic effects of LAs, including lidocaine, have been reported previously. It has been shown that lidocaine has a cytotoxic effect on adipose-derived MSCs during early chondrogenic differentiation and attenuates the fibrogenic, chondrogenic, osteogenic, and adipogenic differentiation of bone marrow–derived MSCs, DPSCs, periodontal ligament stem/progenitor cells, and tendon-derived stem/progenitor cells.^45^, ^46^, ^47^ However, there have been no studies on the effect of lidocaine on the osteogenic differentiation of inflammation-induced MSCs. Therefore, this is the first study to demonstrate that lidocaine attenuates osteogenic differentiation of hDPSCs induced with LPS/TNF-α through the regulation of MAPK pathways. In addition, we demonstrated that the inhibitory effect of lidocaine on the osteogenic differentiation of hDPSCs was not related to cytotoxicity because lidocaine had no cytotoxic effect on the LPS/TNF-α-treated hDPSCs in our experiment.

The present study had some limitations. First, the study was performed in vitro. To apply our results to MSC-based regenerative medicine, further in vivo and clinical studies are needed. Second, the inhibitory effect of lidocaine on the osteogenic differentiation of hDPSCs via the inhibition of ERK and JNK pathways was not confirmed through the use of ERK and JNK activators. If we had confirmed that the degree of staining was less reduced when lidocaine was administered after treatment with ERK and JNK activators in ALP or ARS staining, the ERK and JNK pathways associated with the inhibitory effect of lidocaine on osteogenic differentiation could have been more firmly established.

In summary, we have shown that lidocaine has no cytotoxic effect on hDPSCs and intensified the inhibition of osteogenic differentiation on LPS/TNF-α-treated hDPSCs. In addition, we suggest that the inhibitory effect of lidocaine on the osteogenic differentiation of hDPSCs was mediated via inhibition of the ERK and JNK pathways. Our results indicated that lidocaine can have a negative effect on bone regeneration in bone tissue engineering and regenerative medicine. For more clarification, further studies on the effect of lidocaine on bone regeneration are required.

## Declaration of Competing Interest

The authors have no conflicts of interest relevant to this article.
